# Sex Differences in the Treatment of People with Parkinson’s Disease with a Device-Aided Therapy: A Prospective Real-World Study

**DOI:** 10.3390/medsci14020217

**Published:** 2026-04-27

**Authors:** Diego Santos García, Ángela Solleiro Vidal, Marta Blázquez Estrada, Pablo Mir, Nuria López Ariztegui, Déborah Alonso Modino, Inés Legarda, Alejandro Peral, Rocío García-Ramos, Iria Cabo, Pilar Sánchez Alonso, Jorge Hernández-Vara, Javier Ruíz Martínez, María Álvarez Sauco, Gustavo Fernández-Pajarín, Lydia Vela, Francisco Escamilla Sevilla, Jesús Ramírez Sánchez-Ajofrin, Débora M. Cerdán Santacruz, Guillermo González Ortega, DATs-PD GETM Spanish Registry Group

**Affiliations:** 1 Department of Neurology, Hospital Universitario de A Coruña (HUAC), Complejo Hospitalario Universitario de A Coruña (CHUAC), C/As Xubias 84, 15006 A Coruña, Spain; angela.solleiro@outlook.es; 2 Grupo de Investigación en Enfermedad de Parkinson y Otros Trastornos del Movimiento, INIBIC (Instituto de Investigación Biomédica de A Coruña), 15006 A Coruña, Spain; 3 Hospital San Rafael, 15006 A Coruña, Spain; 4 Fundación Degen, 15006 A Coruña, Spain; 5 Hospital Universitario Central de Asturias (HUCA), 33011 Oviedo, Spain; marta.blazquez.estrada@gmail.com; 6 Unidad de Trastornos del Movimiento, Servicio de Neurología, Instituto de Biomedicina de Sevilla, Hospital Universitario Virgen del Rocío/CSIC/Universidad de Sevilla, 41013 Seville, Spain; pmir@us.es; 7 Centro de Investigación Biomédica en Red sobre Enfermedades Neurodegenerativas (CIBERNED), Instituto de Salud Carlos III, 28031 Madrid, Spain; 8 Departamento de Medicina, Facultad de Medicina, Universidad de Sevilla, 41009 Seville, Spain; 9 Hospital Universitario de Toledo, 45071 Toledo, Spain; nlariztegui@gmail.com; 10 Hospital Universitario de la Candelaria, 38010 Santa Cruz de Tenerife, Spain; deborahalonsomodino@gmail.com; 11 Hospital Universitario Son Espases, 07120 Palma de Mallorca, Spain; ines.legarda@ssib.es; 12 Consorci Sanitari Integral, Hospital Moisés Broggi, Sant Joan Despí, 08970 Barcelona, Spain; aperalq@csi.cat; 13 Hospital Universitario Clínico San Carlos, 28040 Madrid, Spain; garciaramosg@yahoo.es; 14 Complejo Hospitalario Universitario de Pontevedra (CHOP), 36071 Pontevedra, Spain; icabol@yahoo.es; 15 Hospital Universitario Puerta de Hierro, 28222 Madrid, Spain; pisanchezal@gmail.com; 16 Hospital Universitario Vall d’Hebron, 08035 Barcelona, Spain; hernandezvarajorge76@gmail.com; 17 Hospital Universitario Donostia, 20014 San Sebastián, Spain; javier.ruizmartinez@osakidetza.eus; 18 Hospital General Universitario de Elche, 03203 Alicante, Spain; mariaalsa@hotmail.com; 19 Complejo Hospitalario Universitario de Santiago de Compostela (CHUS), 15706 Santiago de Compostela, Spain; gferpaj@gmail.com; 20 Hospital Universitario Fundación de Alcorcón, 28922 Madrid, Spain; lydia.veladesojo@salud.madrid.org; 21 Servicio de Neurología, Hospital Universitario Virgen de las Nieves, Instituto de Investigación Biosanitaria, ibs.GRANADA, 18014 Granada, Spain; fescamilla@hotmail.com; 22 Hospital Universitario 12 de Octubre, 28041 Madrid, Spain; jesufrin96@gmail.com; 23 Hospital General de Segovia, 40002 Segovia, Spain; deboracerdan@hotmail.com; 24 Hospital Universitario de Móstoles, 28935 Madrid, Spain; guilleglez93@gmail.com

**Keywords:** advanced Parkinson’s disease, device-aided therapies, effectiveness, real-world study, safety, sex

## Abstract

**Background and Objective:** Sex differences in the treatment of people with Parkinson’s disease (PwP) with a device-aided therapy (DAT) have been poorly investigated. Our aim was to analyze sex differences in the management and response to a DAT in PwP in daily clinical practice (DCP). **Patients and Methods:** Data collected in the DATs-PD GETM Spanish Registry until 30 October 2025 were used. This is a descriptive, observational, prospective, and multicenter clinical registry with progressive inclusion of PwP treated with a DAT in DCP conditions in more than 40 centers from Spain. Sex differences in the DAT received and changes in quality of life (QoL), motor symptoms (MS), non-motor symptoms (NMS), and autonomy for activities of daily living (AADL) after 6 months of treatment were analyzed. **Results:** A total of 618 PD patients (66.9 ± 9.5 years old; 57.6% men) were treated with a DAT. A significant difference was observed in the DAT type according to sex (*p* = 0.006), with 73.1% of PwP who were treated with deep brain stimulation being men. At the time of DAT indication, women were older, received a higher levodopa equivalent daily dose, and had a worse health-related QoL and AADL. OFF time decreased, whereas MS and NMS burden and health-related QoL improved at 6 months follow-up in both groups (men and women). AADL improved only in men during the OFF state. **Conclusion:** Sex differences were detected in the use of DATs in PwP. Improvement in OFF time, MS, NMS, and QoL was detected in both groups.

## 1. Introduction

Device-aided therapies (DATs) are treatment modalities that utilize medical devices to deliver continuous dopaminergic stimulation or neuromodulation. They include deep brain stimulation (DBS), continuous subcutaneous apomorphine infusion (CSAI), continuous subcutaneous infusion of foslevodopa/foscarbidopa (fLD/fCD), continuous infusion of levodopa–carbidopa intestinal gel (LCIG), and continuous infusion of levodopa–entacapone–carbidopa intestinal gel (LECIG) [1]. DATs are indicated in patients with Parkinson’s disease (PD) with persistent disabling symptoms despite conventional treatment. Selection among these options is individualized based on symptom profile, comorbidities, patient preference, and contraindications [2]. In particular, sex and gender significantly influence both the use and outcomes of DATs in people with PD (PwP). Women are less likely to receive DATs, such as DBS and LCIG, despite similar indications and potential benefits [3,4,5]. This disparity is multifactorial, involving later referral, longer disease duration at intervention, clinical phenotype, and sociocultural barriers, including reduced access to caregiving and healthcare resources [6]. Specifically, women are less likely to receive DBS and are often referred later in the disease course [7]. Motor outcomes after DBS are similar for both sexes, though men may experience greater improvement in bradykinesia and reduction in dopaminergic medication, while women may have more improvement in activities of daily living and cognition but also more mood-related complications postoperatively [8]. No evidence supports preferential use of levodopa intestinal gel (LCIG and LECIG) or continuous subcutaneous infusion therapy (fLD/fCd and CSAI) for either sex, but these therapies may be considered in patients with contraindications to surgery or higher risk of DBS-related complications [9]. Sex disparities in access to DATs persist, with women underrepresented in trials and less likely to be offered advanced therapies (including new fLD/fCD and LECIG), highlighting the need for more research and guidance on sex-specific considerations. Recently, clinical trial data for fLD/fCD demonstrate that a majority of participants were men (68% in the double-blind study [10] and 59.8% in the open-label study [11]). Regarding LECIG, the other recent DAT available, the frequency of men who received the treatment in recently published observational studies was 61.6% [12] and 59% [13]. Moreover, no studies with infusion therapies have reported sex-specific efficacy or safety data. All of these data are consistent with what was observed in a recent meta-analysis that found that women continue to be underrepresented in PD clinical trials, including those for infusion therapies [14]. Effectively addressing this underrepresentation necessitates a holistic approach spanning research, clinical practice, and policy development.

In summary, data on sex-specific preferences for device-aided therapies remain limited, especially regarding new infusion therapies and in gender-diverse populations [15]. The aim of the present study was to analyze the sex differences in the selection, management, and response to a DAT in PwP in daily clinical practice in Spain.

## 2. Material and Methods

Data collected in the DATs-PD GETM Spanish Registry until 30 October 2025 were used. This is a descriptive, observational, prospective, and multicenter clinical registry with progressive inclusion of PwP treated with a DAT in daily clinical practice conditions in more than 40 centers from Spain (Appendix A) [16]. Specifically, the eligibility criteria are [16]: (1) diagnosis of PD according to the MDS criteria [17]; (2) start of treatment with a DAT from 1 January 2024 to; (3) the patient’s desire to participate on a completely voluntary basis; (4) signing of an informed consent.

Data from the baseline visit (V1; before initiation the DAT) and first follow-up visit (V3.6M; 6 ± 3 months after initiation the DAT) were considered for the analysis [16]: sociodemographic data; data about PD (age onset, disease duration, motor phenotype, etc.); comorbidities; treatments; main reason for therapy indication; levodopa equivalent daily dose (LEDD) and dopamine agonist equivalent daily dose (DAEDD) [18]; motor symptoms (MS); non-motor symptoms (NMS), including cognition; Unified Parkinson’s Disease Rating Scale (UPDRS-III) [19] during the OFF and during the ON state; Hoehn & Yahr (H&Y) [20] stage in OFF and in ON; Parkinson’s Disease Questionnaire (PDQ-39; it was expressed as a summary index [PDQ-39SI]) [21]; European Health Interview Survey-Quality of Life 8-item index (EUROHIS-QOL8) [22]; Schwab and England Activities of Daily Living Scale (ADLS) [23] during the OFF and during the ON state. Moreover, a motor symptoms score (MSs) and a non-motor symptoms score (NMSs) ranging from 0 to 60 and from 0 to 80, respectively, were calculated based on 15 MS and 20 NMS collected at each visit (V1 and V3.6M), as it has been previously published [24] (Table S1—Supplementary Material). A specific “Dyskinesia score” (from 0 to 12) was also calculated using the same methodology with 3 items (percentage of the waking day with dyskinesia; dyskinesia severity; painful dyskinesia) [24]. Information was collected on therapy discontinuations and their reasons. However, this study did not specifically analyze aspects related to drug management and complications for each DAT by sex, which will be analyzed together with differences in the selection for each DAT by sex in detail in another manuscript.

Regarding sex, PD patients were classified according to biological aspect in men vs. women. Sex differences in the DAT received and changes in quality of life (QoL), MS, NMS, and autonomy for activities of daily living (AADL) after 6 months of treatment were analyzed.

### 2.1. Data Collection and Statistical Analysis

Data were collected using REDCap (https://project-redcap.org/). Data collected were transferred to a statistical package for subsequent analysis (SPSS 20.0 for Windows). Remote monitoring of the data was carried out (A.S.V.). The sample size was considered according to the data available for each of the analysis variables.

After checking for normality for continuous variables (Shapiro–Wilk’s test), comparisons were carried out intra- and intergroup, applying parametric (Student’s *t*-test for paired or unpaired data) or nonparametric tests (Wilcoxon’s/Mann–Whitney’s tests). Proportions were compared by chi-squared test. To evaluate the magnitude of the change, in addition to the difference between V1 and V3.6M, the relative change [RC  =  mean (TestV3.6M − TestV1) × 100/mean TestV1)] [25] and Cohen’s d effect size [ES  =  mean (TestV3.6M − TestV1)/SD TestV1] [26] were calculated. Cohen’s d effect size was considered to be: absent, <0.2; small, 0.2–<0.5; moderate, 0.5–<0.8; large, 0.8–1.3; or very large, ≥1.3. Specifically, differences between men and women in the change in PDQ-39SI, EUROHIS-QOL8, daily OFF time, MSs, Dyskinesia score, NMSs, ADLS-OFF, and ADLS-ON from V1 to V3.6M were analyzed. The *p*-values were computed using general linear models (GLM) repeated measures without and after adjustment for covariates (age, disease duration, and days receiving the DAT).

### 2.2. Standard Protocol Approvals, Registrations, and Patient Consents

The project was conducted in accordance with the ICH Good Clinical Practice version 6 Revision 2 standard, the fundamental ethical principles established in the Declaration of Helsinki and the Oviedo Convention, as well as the Spanish legal requirements for biomedical research (Biomedical Research Law 14/2007). The Project was approved on 2 April 2024 by the IRB “Comité de Ética de la Investigación Clínica de Galicia from Spain” with code number 2024/109. Written informed consents from all participants in this study were obtained.

### 2.3. Data Availability

The protocol, statistical analysis plan and unidentified participant data will be available on request.

## 3. Results

**Figure 1:**
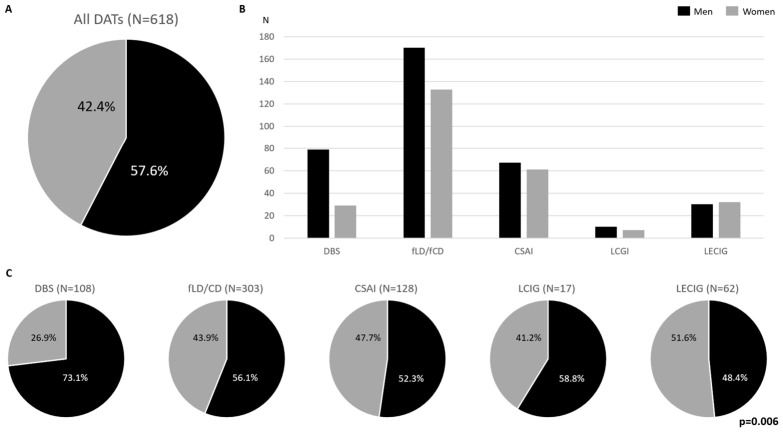
Total frequency (**A**) and specific frequency (**C**) and total number (**B**) of PwP who were treated with a different DATs according to sex.

A total of 618 PD patients were treated with a DAT. The mean age was 66.9 ± 9.5 years old, with 356 (57.6%) being men and 262 (42.4%) being women. Regarding the DAT type, the frequency was 49.0% (N = 303) fLD/fCD, 20.7% (N = 128) CSAI, 17.5% (N = 108) DBS, 10% (N = 62) LECIG, and 2.8% (N = 17) LCIG. The frequency of subcutaneous therapies (fLD/fCD + CSAI) was 69.7% versus 12.8% for enteral therapies (LECIG + LCIG). A significant difference was observed in the DAT type according to sex (*p* = 0.006) (Figure 1C). Specifically, 79 men (73.1%) were treated with DBS compared with 29 (26.9%) women. Regarding the rest of the DATs, the number of subjects who were treated with a DAT was greater in men vs. women (fLD/fCD, 170 vs. 133 CSAI, 67 vs. 61; LCIG, 10 vs. 7) except in the case of LECIG (30 vs. 32) (Figure 1B).

At baseline before DAT initiation (V1), women were older than men (69.2 ± 9.8 vs. 65.2 ± 8.9; *p* < 0.0001). Smoking, alcohol consumption, ischemic cardiopathy, hyperuricemia, and cancer were more frequent in men than in women (Table 1). The percentage of men with a principal caregiver was lower for men compared to women (50.3% vs. 59%; *p* = 0.020), with the partner being the principal caregiver in 81.2% of men compared to 64% of women (*p* < 0.0001; Table 1). Compared with women, men were being treated more frequently with a type-B monoamine oxidase inhibitor (56% vs. 44.3%; *p* = 0.003) and with a catechol-O-methyltransferase inhibitor (58.5% vs. 50.2%; *p* = 0.027), being the LEDD and the DAEDD significantly higher in men vs. women (Table 2). Significant difference between both groups was detected in weight (*p* < 0.0001) but not in the BMI (body mass index) (Table 2). Regarding cognitive impairment, more men were cognitively intact compared with women (78.5% vs. 69.5%; *p* = 0.045). With reference to PD symptoms, mean daily OFF time was 5.5 ± 3.1, without differences between both groups (*p* = 0.907) (Table 2). No differences were also found in time with fluctuations, H&Y, UPDRS-III, MSs, and NMs, but so in Dyskinesia score, being 3.0 ± 2.2 in women compared to 2.5 ± 2.0 in men (*p* = 0.002) (Table 2). In terms of QoL and AADL, women compared to men had at baseline a worse health-related QoL (PDQ-39SI, 42.8 ± 14.3 vs. 34.6 ± 15.5; *p* < 0.0001) and a lesser AADL during the ON state (ADLS-ON, 77.9 ± 18.7 vs. 82.9 ± 17.2; *p* < 0.0001). Specifically, 65.4% of the men were independent for activities of daily living compared to 54.1% of the women (*p* = 0.029) (Table 2).

**Figure 2:**
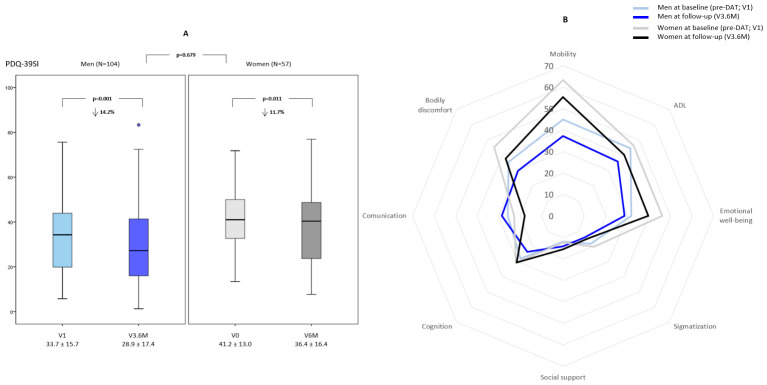
(**A**) Change from the baseline visit (pre-DAT; V1) to the follow-up visit at 6 ± 3 months (V3.6M) in the PDQ-39SI in men vs. women. Data are presented as box plots, with the box representing the median and the two middle quartiles (25–75%). Mild outliers (O) are data points that are more extreme than Q1—1.5. Nonparametric tests were applied. (**B**) Mean value of each domain of the PDQ-39 at V1 and at V3.6M in men (in blue) vs. women (in gray). Nonparametric tests were applied.

The mean follow-up time in the total cohort (N = 313) was 188.6 ± 72.9 days (men [N = 199] 186.7 ± 65.2 days; women [N = 114] 192.0 ± 85.0; *p* = 0.659). PwP improved from V1 to V3.6M in daily OFF time (RC = −63.5%; Cohen’s d = 1.14; *p* < 0.0001), dyskinesia (Dyskinesia score; RC = −38.5%; Cohen’s d = 0.43; *p* < 0.0001), MS (MSs; RC = −41.6%; Cohen’s d = 1.16; *p* < 0.0001), NMS (NMSs; RC = −27.1%; Cohen’s d = 0.50; *p* < 0.0001), health-related QoL (PDQ-39SI; RC = −13.4%; Cohen’s d = 0.34; *p* < 0.0001), and AADL during the OFF state (ADLS-OFF; RC = +8.3%; Cohen’s d = 0.23; *p* = 0.001) (Table 3). According to sex, daily OFF time decreased significantly in both groups without differences, with a reduction of 3.2 ± 2.9 h in men (RC = −62.7%; Cohen’s d = 1.10; *p* < 0.0001) and 3.6 ± 2.9 h in women (RC = −67.9%; Cohen’s d = 1.24; *p* < 0.0001). From V1 to V3.6M, the Dyskinesia score, MSs, NMSs, and PDQ-39SI decreased significantly in both men and women, whereas the EUROHISQOL8 and ADLS-OFF increased significantly only in men (Table 3). After adjustment for age, disease duration, and days receiving the DAT, there was a significant difference in the change in Dyskinesia score from V1 to V6.M between both groups (from 2.5 ± 2.0 to 1.3 ± 1.7 in men vs. from 2.9 ± 2.3 to 2.1 ± 1.8 in women; *p* = 0.002), with a specific difference in the RC of 20.4% in favor of men (*p* = 0.006). Regarding QoL, although significant differences were detected between men and women in the change in the PDQ-39SI (*p* = 0.005) and the EUROHISQOL8 (*p* = 0.035) from V1 to V3.6M (Table 3) after adjustment to covariates, no significant differences were detected in the RC for both PDQ-39SI (−14.2% in men vs. −11.7% in women [*p* = 0.679]) (Figure 2A) and EUROHISQOL8 (+6.0% in men vs. +0.6% in women [*p* = 0.188]). According to the domains of the PDQ-39, only differences in the RC between men and women were detected in cognition (−15.7% in men vs. −1.2% in women; *p* = 0.023) and communication (−1.1% in men vs. −21.1%; Table 4 and Figure 2B). No other differences in the GLM repeated measures after adjustment to covariates were detected between groups (men vs. women) in other aspects.

In accordance with the data of the DATs-PD GETM Spanish Registry for this (N = 618) cohort until 30 October 2025, 101 out of 618 (16.3%) PwP discontinued with the therapy: 4/13 (23.5%) LCIG, 25/128 (19.5%) CSAI, 59/303 (19.5%) fLD/fCD, 11/62 (17.7%) LECGI, and 2/108 (1.9%) DBS. After excluding DBS, the rate of discontinuation of the DATs–pump group was 99/510 (19.4%). No differences were found in the discontinuation of the DAT, being 15.4% in men (55/356) and 17.6% (46/262) (*p* = 0.484). The percentage was 19.1% in men and 19.7% in women when only a pump system was considered (*p* = 0.863). The reasons for the discontinuation of the DAT were any complication related to the therapy (N = 47; 7.6%), a direct decision of the patient and/or principal caregiver (N = 20; 3.2%), another reason (N = 33; 5.3%), and loss of follow-up (N = 1; 0.2%). In the group who discontinued the DAT (N = 101), the frequency of discontinuation due to a direct decision of the patient and/or principal caregiver was similar in both sexes, 20% in men vs. 19.6% in women, but discontinuation due to any complication related to the therapy had a trend to be significantly higher in women than in men (58.7% vs. 36.4%; *p* = 0.085). Ten patients (1.6%) died for different reasons (two with infection with sepsis; two with cancer; one with intestinal perforation; one with gastrointestinal bleeding; four with no reported cause), all of them being men except one woman.

## 4. Discussion

The present study observed relevant differences in the management of advanced PD concerning the treatment with a DAT in a Spanish cohort of more than 600 PwP in relation to sex. Specifically, women were less frequently treated with a DAT, which is very noticeable in the case of DBS (less than one in four patients), and when they were treated, they were older, more frequently had cognitive impairment and a worse health-related QoL, and had lesser AADL. Of note, this study analyzing differences in DATs by sex has the largest sample size to date and includes for the first time recently emerged therapies, fLD/fCD and LECIG. Furthermore, and in line with recent studies [24], fLD/fCD treatment was the most used DAT in both sexes, accounting for almost half of all the cases, while enteral therapies only accounted for 13%, which reinforces the idea that subcutaneous therapies could be the first choice in many cases due to their lower cost, invasiveness, and complexity of implantation.

Previously published data indicate that there are sex differences in many features of PD [27,28,29,30,31,32,33]. A recent meta-analysis study found, using pooled data of 4352 PD patients (58% men), that women showed higher symptom severity in mood/cognition, whereas higher severity in the sexual domain was found in men, highlighting the need for a specific sex-related approach in PD [32]. In agreement with the literature, a study conducted in Spanish PwP (N = 681) showed that symptoms such as depression, fatigue, and pain were more frequent and/or severe in women, whereas other symptoms, such as hypomimia, speech problems, rigidity, and hypersexuality, were more noted in men [33]. However, sex differences appear to exist in access to DATs as well, with differences about the indication and management of the DAT being poorly studied [3,4,5], and particularly, without previously published data in Spain. The findings observed in this study are consistent with the idea that women receive a DAT for PD significantly less frequently than men. In a large Medicare study, women were less likely to receive any DAT compared to men, even after controlling for disease severity [34]. Different studies have found a male predominance for DBS and LCIG [3,4,5,34,35,36,37,38,39,40,41,42]. In our cohort, the greatest difference was observed in DBS, being 73% of PWP-treated men. Women represent only 27–30% of DBS recipients despite comprising approximately 40% of the PD population [35,36]. This underrepresentation persists across multiple countries and healthcare systems [37,38]. Women are referred less frequently for DBS evaluation (relative risk 0.72) and have a 26% lower overall likelihood of undergoing DBS treatment [36]. However, when referred, women are more likely to be approved for DBS than men (relative risk 1.17), suggesting the primary barrier is at the referral stage rather than candidacy. While men may tend to have a decision-making process driven by their own initiative, women tend to hesitate and wait, being more anxious and appearing more fearful about complications [8]. LCIG shows less sex disparity than DBS, with men comprising about 55–65% of LCIG recipients across multiple studies [38,41,42], but more disparity than CSAI, where women approached or exceeded parity with men [38,43,44]. Several factors may contribute to the more balanced sex distribution with CSAI, such as less invasive and simpler initiation than DBS and LCIG, pharmacokinetic equivalence, and smaller sample sizes in published studies, which may limit the generalizability of these findings. However, our findings, with 52.3% men out of 128 patients receiving CSAI, support these data. Although there is less evidence on the use of the new therapies, fLD/fCD and LECIG, data from some studies may suggest a men predominance similar to LCIG [11,12,13,14]. However, comparison by sex continues to be largely ignored, to the point that some recent observational studies of fLD/fCD do not provide even any information on the sex/gender variable [45,46]. In this cohort, and contrary to other observational studies [13,14], LECIG was the only DAT with more women treated than men. Another factor that could influence the decision is whether there is a principal caregiver and who he/she is. In this study, the difference between groups in the caregiver, with men more frequently having a caregiver who was their spouse, could have also influenced the decision, although this is something that was not explored as such. Previous studies observed that male patients may receive more intensive caregiving than women patients [47]. Data from a larger number of patients from this registry [16] will give us more certainty in the future about the frequency of use of DATs according to sex. Furthermore, to know for sure whether more men than women are treated with a DAT, a population study would be necessary that specifically analyzes the number of cases of each sex treated with respect to the total number of patients of each sex with PD in that area.

An essential point is that women referred for DATs present with more advanced disease compared to men, suggesting the women may be referred later in their disease course, potentially missing the optimal therapeutic window [38,39]. However, studies investigating sex disparity in the access to fLD/fCD, CSAI, LCIG, and LECIG are lacking, with only a few focusing on DBS [3,5,8,34,35,36,37]. At the time of DBS evaluation, women demonstrate significantly longer disease duration, worse motor symptoms (higher UPDRS-III off-medication scores), and lower QoL scores despite receiving lower LEDD [3,5,8,34,35,36,37]. These findings agree with ours for this cohort, in which all DATs are considered as a whole. On the contrary, in 2024 Maccarrone et al. reported, in a study in which PwP treated with DBS, CSAI, LCIG, and MrgFUS thalamotomy were included, no differences in age, disease duration, H&Y and the previous LEDD between men and women [38]. Large observational studies of LCIG have included both men and women but generally have not reported sex-disaggregated outcomes. For example, the TANDEM study included 55% men among 159 patients and demonstrated significant improvements in motor scores and complications, but did not analyze outcomes by sex [48]. Similarly, the GREENFIELD study and COSMOS long-term follow-up studies included mixed-sex cohorts but did not report sex-specific efficacy or safety data [49,50]. This same circumstance occurs in the case of CSAI and the new fLD/fCD and LECIG therapies. In the present study, women showed at baseline a greater affectation in dyskinesia, representing this sex difference as one of the most clinically important disparities in PD treatment complications [27,28,51]. Furthermore, both groups (men and women) improved in dyskinesia, but this improvement was reduced in women, as has been previously reported with DBS in some studies [52]. In future analyses in this cohort, it will be of interest to analyze the differences in dyskinesias between men and women according to each DAT and the influence of important variables such as weight, LEDD, sex differences in levodopa pharmacokinetics, and higher baseline dyskinesia severity in women. OFF time, MS and NMS burden, and health-related QoL improved after 6 months with a DAT in each group (men and women) similarly to what has been reported in the literature in general populations of PwP, but again, there is a lack of studies on sex differences [5,8,14,37,38,39,40,41,42,43,44,45,46,48,49,50,51]. Specifically, regarding QoL, women were more affected before treatment, as in other studies [3,36], and both men and women improved, but no percentage differences were detected between the two groups. A 2024 systematic review [8] concluded that, regarding QoL, the evidence for DBS seems to be inconsistent, with activities of daily living improving in both sexes but conflicting data on overall QoL measures. Again, no published studies have performed sex-stratified analyses of QoL outcomes with fLD/fCD, CSAI, LCIG, and LECIG. In this context, it is absolutely necessary to conduct studies analyzing the differences by sex in the selection of candidates for a DAT as well as the response and outcome, including the analysis individually by DAT.

This study observes that in clinical practice there are sex differences in the indication of DATs in patients with PD. From a practical standpoint, it would be advisable to investigate the causes of these findings and attempt to correct them if they result in inequity in decision making. For example, it is important to invest time in explaining to the patient the realistic goals of a possible benefit with the DAT and also the possible complications if, for example, the fear of a side effect may be a more frequent reason for rejection in one sex than the other. In other words, it is not only important to analyze the differences [38] but also their causes and to propose strategies to improve management. A study specifically designed to analyze this on a large scale would be of utmost interest.

The present study has some limitations. First, although the overall sample size is relatively large for a study with this type of treatment (i.e., DATs), it varies between groups, resulting in a small sample size for enteral therapies. Second, the analysis of differences by sex has been carried out considering all DATs as a whole, without going into detail on the differences by sex in each of them. Third, the follow-up period is short, making it necessary to observe medium- to long-term outcomes. Fourth, this study did not analyze the complications experienced by patients or the specific management of DATs and other treatments for PD. This will be addressed in a second subsequent analysis. Fifth, although some validated scales and/or questionnaires were used (H&Y, UPDRS, ADLS, PDQ-39, etc.), some scores defined to measure other symptoms [16,24] lack validation. Moreover, information about a specific cognitive test score was not provided. Sixth, there are all of the limitations inherent to the methodology used, which is the extraction of data from a registry, such as not completing all variables in all cases. Finally, despite the indication of consecutive inclusion of PwP treated with a DAT in the hospitals participating from Spain, this was not fulfilled, and there is an inclusion bias. For all of these reasons, the results should be interpreted with extreme caution and await corroboration in other well-planned studies.

In conclusion, we report here the first study to date in which the differences by sex in the characteristics of PwP selected to be treated with a DAT, as well as their response at 6 months, were analyzed. Women were less frequently treated with a DAT than men, especially in the case of DBS with a higher percentage of men (>70%) than the known prevalence ratio of approximately 60 vs. 40% (men vs. women). When PD patients are treated with a DAT, women are more affected by PD. Despite this, OFF time, MS and NMS burden, and health-related QoL improve in both groups. There is a striking lack of evidence on this topic and more studies are needed.

## Figures and Tables

**Table 1:** Sociodemographic and aspects related to comorbidities in PwP at baseline (before initiation with a DAT) in men vs. women from the DATs-PD GETM Spanish registry.

	All Cohorts N = 618	Men N = 356	Women N = 262	*p*
Civil status (%):				**<0.0001**
-Married	72.7	78.1	65.5	
-Single	10.3	11.7	8.5	
-Widowed	9.7	4.3	17.1	
-Other	7.3	5.9	5.9	
Living style (%):				**<0.0001**
-With the partner	73.9	81.2	64	
-Alone	10.5	9.7	11.6	
-With a son/daughter	7.7	3.1	14	
-Institutionalized	2	0.6	3.9	
-Other	5.9	5.4	6.7	
Principal caregiver (%):	54	50.3	59	**0.020**
Caregiver (%):				**<0.0001**
-Partner	67.7	80	53.6	
-Son/daughter	16.8	9.1	25.5	
-Other	15.5	10.9	20.9	
Smoking (%)				**<0.0001**
-Active	6.8	7.5	5.8	
-Ex-smoker	30.2	27.3	10.8	
-Non-smoker	73	65.2	83.4	
Alcohol (%)	24.4	32.6	13.3	**<0.0001**
Comorbidities (%):				
-Arterial hypertension	37.5	37.7	37.3	0.926
-Diabetes mellitus	14.7	17.1	11.5	0.055
-Dyslipemia	31.5	31.7	31.3	0.905
-AF/arrhythmia	7	6.2	8.1	0.375
-Ischemic cardiopathy	4.6	6.8	1.5	**0.002**
-Lung disease	7.8	8.5	6.9	0.467
-Renal disease	2.8	3.1	2.3	0.541
-Polyneuropathy	2.5	1.7	3.5	0.166
-Hyperuricemia	3.3	5.1	0.8	**0.003**
-Cancer				**0.011**
Previous	8.4	9.1	7.3	
Active	1.8	3.1	0	
Treatments (%):				
-Antihypertensive	34.8	36.7	32.3	0.263
-Antidiabetics	14	16	11.2	0.341
-Hypolipidemic agents	25.9	27.8	23.2	0.192
-Antidepressant	35.9	32.3	40.9	0.561
-Benzodiazepine	36.1	35.7	36.7	0.806
-Antipsychotic	12.4	13.8	10.3	0.569
-Anti-dementia	7.3	6.2	8.8	0.750

The results represent percentages; *p*, comparison between both groups, men vs. women. Chi-square test was applied. AF, atrial fibrillation.

**Table 2:** PD-related variables at baseline (before initiation with a DAT) in men vs. women from the DATs-PD GETM Spanish registry.

	All Cohorts N = 618	Men N = 356	Women N = 262	*p*
Age at initiation of DAT	66.9 ± 9.5	65.2 ± 8.9	69.2 ± 9.8	**<0.0001**
Years since PD diagnosis	11.2 ± 6.9	10.7 ± 5.6	11.8 ± 8.3	0.490
Motor phenotype (%):				0.910
-Tremor dominant	34.2	33.4	35.2	
-Indeterminate	31.6	32.1	30.9	
-PIGD	34.2	34.5	33.9	
				
Treatment for PD (%):				
-MAO-B inhibitor	65.5	65.4	65.6	0.513
-COMT inhibitor	51	56	44.3	**0.003**
-Dopamine agonist	55	58.5	50.2	**0.027**
-Amantadine	27.5	26.5	28.7	0.306
Previous ODT	47	47.4	46.3	0.424
Previous DAT	23.5	24	22.9	0.411
LEDD (mg)	1193.7 ± 559.2	1293.8 ± 606.9	1064.6 ± 461.0	**<0.0001**
DAEDD (mg)	106.5 ± 126.1	113.2 ± 123.3	97.5 ± 129.6	**0.033**
Weight	72.5 ± 14.9	78.8 ± 12.5	64.0 ± 13.6	**<0.0001**
BMI	26.8 ± 4.1	27.1 ± 3.2	26.1 ± 5.4	0.414
				
Previous psychosis (%)	10.3	8.8	12.4	0.101
Cognitive impairment (%):				**0.045**
-MCI (%)	22.8	19.5	27.3	
-Dementia (%)	2.5	2	3.2	
				
Time with fluctuations (y)	5.2 ± 3.6	4.9 ± 3.3	5.5 ± 3.9	0.285
H&Y—OFF	3 [2, 4]	3 [2, 4]	3 [2, 4]	0.249
H&Y—ON	2 [2, 2]	2 [2, 2]	2 [2, 3]	0.073
UPDRS—III—OFF	38.7 ± 12.7	38.8 ± 12.7	38.5 ± 13.4	0.792
UPDRS—III—ON	20.9 ± 11.4	20.8 ± 11.7	21.0 ± 11.0	0.858
Daily OFF time (h)	5.5 ± 3.1	5.5 ± 3.0	5.5 ± 3.1	0.907
MSs	15.5 ± 6.0	15.4 ± 6.1	15.7 ± 5.9	0.560
Dyskinesia score	2.7 ± 2.1	2.5 ± 2.0	3.0 ± 2.2	**0.002**
NMSs	12.2 ± 7.1	11.9 ± 7.1	12.7 ± 7.1	0.142
				
PDQ-39SI	37.9 ± 25.6	34.6 ± 15.5	42.8 ± 14.3	**<0.0001**
EUROHIS-QOL8	22.3 ± 5.0	22.7 ± 5.2	21.8 ± 4.7	0.093
ADLS—OFF	56.6 ± 24.0	57.9 ± 24.1	54.6 ± 23.8	0.122
ADLS—ON	80.8 ± 17.9	82.9 ± 17.2	77.9 ± 18.7	**<0.0001**
Daily living activities (%):				**0.019**
-Independence	60.6	65.4	54.1	
-Dependence for IADL	23.9	20.6	28.4	
-Dependence for BADL	15.5	14	17.5	

The results represent percentages, mean ± SD or median [p25, p75]; *p*, comparison between both groups, men vs. women. Chi-square, Student t and/or Mann–Whitney/Wilcoxon tests were applied according to the type of analysis and distribution of the variables. The information was not collected for all patients. The lowest sample size was N = 43 (6.9%) for BMI, N = 333 (53.9%) for weight, N = 376 for UPDRS-III-ON (60.8%), N = 386 for EUROHIS-QOL8 (62.5%), N = 395 for PDQ-39SI (63.9%), N = 486 for UPDRS-III-OFF (78.6%), N = 494 (79.9%) for ADLS—OFF, and N = 500 (80.9%) for ADLS—OFF; the sample was >90% for the rest of the variables. MSs, NMSs, and Dyskinesia score were calculated according to previous publications (Santos-García et al. Mov Disord Clin Pract 2025 [15]; Santos-García et al. J Neural Transm 2025 [24]). The *p*-values < 0.05 are shown in bold. ADLS, Schwab and England Activities of Daily Living Scale; BADL, basic activities of daily living; BMI, body mass index; COMT, catechol-O-methyltransferase; DAEDD, dopamine agonist equivalent daily dose; DAT, device-aided therapy; EUROHIS-QOL8, European Health Interview Survey-Quality of Life 8-item index; H&Y, Hoehn & Yahr; IADL, instrumental activities of daily living; LEDD, levodopa equivalent daily dose; MAO-B, type-B monoamine oxidase; MCI, mild cognitive impairment; MSs, motor symptoms score; NMSs, non-motor symptoms score; ODT, on-demand therapy (i.e., inhaled levodopa or apomorphine); PDQ-39SI, 39-item Parkinson’s Disease Quality of Life Questionnaire; PIGD, postural instability gait difficulty; UPDRS, Unified Parkinson’s Disease Rating Scale.

**Table 3:** Comparison between men and women treated with a DAT in the change from the baseline to the final visit at 6 months in the main variables analyzed related to PD.

	All Cohorts	Men	Women	*p*_a_
**Daily OFF time (h)**				0.693 0.724
N	264	169	95	
V1	5.2 ± 2.7	5.1 ± 2.8	5.3 ± 2.8	
V3.6M	1.9 ± 1.9	1.9 ± 2.0	1.6 ± 1.8	
OFF time reduction (h)	3.3 ± 2.9	3.2 ± 2.9	3.6 ± 2.9	
Relative change %	−63.5	−62.7	−67.9	
Cohen’s effect size	1.14	1.10	1.24	
*p*-value (change)	**<0.0001**	**<0.0001**	**<0.0001**	
**Dyskinesia score**				**0.001** **0.002**
N	203	191	112	
V1	2.6 ± 2.1	2.5 ± 2.0	2.9 ± 2.3	
V3.6M	1.6 ± 1.7	1.3 ± 1.7	2.1 ± 1.8	
Relative change %	−38.5	−48.0	−27.6	
Cohen’s effect size	0.43	0.51	0.31	
*p*-value (change)	**<0.0001**	**<0.0001**	**0.001**	
**MSs**				0.272 0.795
N	289	179	110	
V1	15.4 ± 6.1	15.1 ± 6.2	15.9 ± 5.9	
V3.6M	9.0 ± 5.3	8.9 ± 5.1	9.3 ± 5.5	
Relative change %	−41.6	−41.1	−41.5	
Cohen’s effect size	1.16	1.05	1.19	
*p*-value (change)	**<0.0001**	**<0.0001**	**<0.0001**	
**NMSs**				0.514 0.878
N	293	182	111	
V1	11.4 ± 6.8	11.1 ± 6.6	11.9 ± 7.2	
V3.6M	8.3 ± 5.8	8.3 ± 5.7	8.4 ± 6.0	
Relative change %	−27.1	−25.4	−29.7	
Cohen’s effect size	0.50	0.49	0.52	
*p*-value (change)	**<0.0001**	**<0.0001**	**<0.0001**	
**PDQ-39SI total score**				**0.001** **0.005**
N	161	104	57	
V1	36.6 ± 15.3	33.7 ± 15.7	41.2 ± 13.0	
V3.6M	31.7 ± 17.4	28.9 ± 17.4	36.4 ± 16.4	
Relative change %	−13.4	−14.2	−11.7	
Cohen’s effect size	0.34	0.33	0.35	
*p*-value (change)	**<0.0001**	**0.001**	**0.011**	
**EUROHIS-QOL8**				0.072 **0.035**
N	161	100	61	
V1	23.3 ± 4.8	23.4 ± 4.8	22.9 ± 4.7	
V3.6M	24.2 ± 5.2	24.8 ± 5.6	23.1 ± 4.4	
Relative change %	+3.9	+6.0	+0.6	
Cohen’s effect size	0.15	0.21	0.03	
*p*-value (change)	0.055	**0.039**	0.792	
**ADLS—OFF**				0.152 0.707
N	238	152	86	
V1	58.2 ± 24.8	59.6 ± 25.1	55.7 ± 24.2	
V3.6M	63.0 ± 23.2	64.6 ± 22.6	60.1 ± 24.1	
Relative change %	+8.3	+8.4	+7.8	
Cohen’s effect size	0.23	0.25	0.18	
*p*-value (change)	**0.001**	**0.002**	0.088	
**ADLS—ON**				**0.020** 0.238
N	236	151	85	
V1	82.9 ± 17.0	84.8 ± 15.2	79.4 ± 19.5	
V3.6M	84.2 ± 16.5	85.7 ± 15.5	81.5 ± 18.1	
Relative change %	+1.5	+0.9	+2.4	
Cohen’s effect size	0.08	0.05	0.12	
*p*-value (change)	0.204	0.464	0.287	

The results represent mean ± SD at V1 and at V3.6M, relative change (%), and Cohen’s effect size. To calculate *p*-value change (from V1 to V3.6M), *p*-values were computed using general linear models (GLM) repeated measures; *p*_a_, comparison between men vs. women. First, the unadjusted *p*-value for covariates is shown, and below, the *p*-value after adjustment for covariates (age, disease duration, and days receiving the DAT). The *p*-values < 0.05 are shown in bold. ADLS, Schwab and England Activities of Daily Living Scale; DAT, device-aided therapy; MSs, motor symptoms score; NMSs, non-motor symptoms score; PDQ-39SI, 39-item Parkinson’s Disease Quality of Life Questionnaire. The *p*-values < 0.05 are shown in bold.

**Table 4:** Relative change in the PDQ-39SI domains from baseline (V1) to follow-up visit (6 ± 3 months [V3.6M]) in men vs. women PD patients from the DATs-PD GETM Spanish registry.

RC % (V6M vs. V1)	All Cohorts N = 161	Men N = 104	Women N = 57	*p*
Mobility	−14.9	−16.9	−12.6	0.236
ADL	−17.1	−19.1	−13.4	0.802
Emotional well-being	−11.7	−9.7	−14.1	0.697
Stigmatization	−22.7	−21.9	−24.6	0.951
Social support	+16.4	+18.3	+25	0.604
Cognition	−10.3	−15.7	−1.2	**0.023**
Communication	−0.4	−1.1	−21.1	**0.014**
Bodily discomfort	−16.6	−16.5	−16.6	0.847

Mann–Whitney/Wilcoxon tests were applied; *p*, comparison between both groups, men vs. women. ADL, activities of daily living. The *p*-values < 0.05 are shown in bold.

## Data Availability

The protocol, statistical analysis plan and unidentified participant data will be available on request.

## Supplementary Materials

The following supporting information can be downloaded at: https://www.mdpi.com/article/10.3390/medsci14020217/s1, Table S1: Supplementary Material. MSs and NMSs used for this study.

## Abbreviations

AADL, autonomy for activities of daily living; CSAI, continuous subcutaneous apomorphine infusion; DATs, device-aided therapies; DBS, deep brain stimulation; fLD/fCD, foslevodopa/foscarbidopa; LCIG, levodopa–carbidopa intestinal gel infusion; LECIG, levodopa–carbidopa–entacapone intestinal gel; LEDD, levodopa equivalent daily dose; MS, motor symptoms; MSs, motor symptoms score; NMS, non-motor symptoms; NMSs, non-motor symptoms score; PDQ-39, 39-item Parkinson’s Disease Questionnaire; PD, Parkinson’s disease; PwP, people with PD; UPDRS, Unified Parkinson’s Disease Rated Scale.

## Appendix A

DATs-PD GETM Spanish Registry STUDY GROUP (only members who participated with available data for this analysis are here included)

Adarmes Gómez AD, Aldaz A, Alonso Modino D, Álvarez Sauco M, Ávila Rivera A, Baviera-Muñoz R, Belmonte S, Blázquez Estrada M, Caballero Sánchez L, Caballol Pons N, Cabo I, Campins Romeu M, Campo Caballero D, Cantarero Duque S, Cañada Lahoz E, Carmona Abellán M, Carrillo García F, Casas Peña E, Castaño García B, Castellano Guerrero AM, Castrillo Sanz A, Cerdán Santacruz DM, Cores Bartolomé C, Cots Foraster A, Cubo Delgado E, del Álamo Criado C, Delgado Ballestero T, Escalante Arroyo S, Escamilla Sevilla F, Fanjul Arbos S, Feliz Feliz C, Fernández-Pajarín G, Fernández Revuelta A, Fernández Valle T, Freire Álvarez E, Gamo González E, García Fernández C, García Herruzo A, García-Ramos R, García Ruíz Espiga P, Garrote Espina L, Gil Villar MP, Gómez Esteban JC, Gómez López de San Román C, Gómez Mayordomo V, Gómez Rapela C, González MV, González Ardura J, González-Ortega G, González Rojas N, Hernández Javier C, Hernández-Vara J, Jesús Maestre S, Legarda I, López Ariztegui N, López Blanco R, López Dominguez D, López Manzanares L, López Valdés E, Lorenzo Barreto P, Lorenzo Brito JM, Lozano D, Lozano Veiga S, Macías García D, Mandrá MA, Madrid Navarro CJ, Martí Martínez S, Martín García R, Martínez Castrillo JC, Martínez-Torres I, Mata Álvarez Santullano M, Mauri Fabrega L, Méndez Guerrero A, Mendoza Rodríguez A, Mir P, Mondragón Rezola E, Monterde Ortega A, Morales Casado MI, Morata-Martínez C, Muñoz Delgado L, Muro I, Navarro Mocholi E, Novo Ponte S, Ojeda Lepe E, Pardina Vilella L, Pareés Moreno I, Paz González JM, Peral A, Pérez Calvo C, Pérez Rangel D, Perona Moratalla A, Planas-Ballvé A, Prendes Fernández P, Quibus Requena L, Rábano Suárez P, Ramírez Sánchez-Ajofrin J, Rivero de Aguilar Pensado A, Rojas Pérez E, Ribacoba Díaz C, Romero Fábrega JC, Ruiz López M, Ruíz Martínez J, Samaniego Vinueza LB, San Eufrasio Martínez M, Sánchez Alonso P, Sánchez Ferro A, Sánchez Rodríguez A, Sancho Saldana A, Santos-García D, Sastre Bataller I, Solano Vila B, Solleiro Vidal A, Suárez San Martín E, Tabar Comellas G, Tijero Merino B, Triguero Cueva L, Valero García MF, Vela L, Vinagre Aragón A, Vivas Villacampa L, and Vives Pastor B.

**First Name**	**Last Name**	**Center**
Diego	Santos García	Complejo Hospitalario Universitario de A Coruña
Jose Manuel	Paz González	Complejo Hospitalario Universitario de A Coruña
Carlos	Cores Bartolomé	Complejo Hospitalario Universitario de A Coruña
Lucia Belen	Samaniego Vinueza	Complejo Hospitalario Universitario de A Coruña
Ángela	Solleiro Vidal ^2^	Complejo Hospitalario Universitario de A Coruña
María	Álvarez Sauco	Hospital General Universitario de Elche
Eric	Freire Álvarez	Hospital General Universitario de Elche
Juan Carlos	Martínez Castrillo	Hospital Universitario Ramón y Cajal
Isabel	Pareés Moreno	Hospital Universitario Ramón y Cajal
Samira	Fanjul Arbos	Hospital Universitario Ramón y Cajal
Ana Belén	Perona Moratalla	Complejo Hospitalario Universitario de Albacete
Inés	Legarda	Hospital Universitario Son Espases
Bàrbara	Vives Pastor	Hospital Universitario Son Espases
María Fuensanta	Valero García	Hospital Universitario Son Espases
Esther	Cubo Delgado	Hospital Universitario de Burgos
Nuria	López Ariztegui	Hospital Universitario de Toledo
Maria Isabel	Morales Casado	Hospital Universitario de Toledo
Guillermo	Tabar Comellas	Hospital Universitario de Toledo
Déborah	Alonso Modino	Hospital Universitario de la Candelaria
Jesús Norelis	Lorenzo Brito	Hospital Universitario de la Candelaria
Esther	Rojas Pérez	Hospital Universitario de la Candelaria
Carolina	Hernández Javier	Hospital Universitario de la Candelaria
Iria	Cabo	Complejo Hospitalario Universitario de Pontevedra
Alejandro	Rivero de Aguilar Pensado	Complejo Hospitalario Universitario de Pontevedra
Jorge	Hernández-Vara	Hospital Universitario Vall d’Hebron
Maria Victoria	González	Hospital Universitario Vall d’Hebron
Sara	Belmonte ^1^	Hospital Universitario Vall d’Hebron
Juan Carlos	Romero Fábrega	Hospital Universitario Virgen de las Nieves
Francisco	Escamilla Sevilla	Hospital Universitario Virgen de las Nieves
Lucía	Triguero Cueva	Hospital Universitario Virgen de las Nieves
Carlos Javier	Madrid Navarro	Hospital Universitario Virgen de las Nieves
Asunción	Ávila Rivera	Complex Hospitalari Moisès Broggi
Núria	Caballol Pons	Complex Hospitalari Moisès Broggi
Anna	Planas-Ballvé	Complex Hospitalari Moisès Broggi
Alejandro	Peral	Complex Hospitalari Moisès Broggi
Dolors	Lozano	Complex Hospitalari Moisès Broggi
Álvaro	Sánchez Ferro	Hospital 12 de Octubre
Pablo	Rábano Suárez	Hospital 12 de Octubre
Antonio	Méndez Guerrero	Hospital 12 de Octubre
Daniel	Pérez Rangel	Hospital 12 de Octubre
Jesús	Ramírez Sánchez-Ajofrin	Hospital 12 de Octubre
Cristina	del Álamo Criado	Hospital 12 de Octubre
Rocío	García-Ramos	Hospital Clínico Universitario San Carlos
Ana	Fernández Revuelta	Hospital Clínico Universitario San Carlos
Eva	López Valdés	Hospital Clínico Universitario San Carlos
Carmen	Ribacoba Díaz	Hospital Clínico Universitario San Carlos
Ana	Aldaz	Hospital Clínico Universitario San Carlos
Irene	Martínez-Torres	Hospital Universitario la Fe
Carlos	Morata-Martínez	Hospital Universitario la Fe
Raquel	Baviera-Muñoz	Hospital Universitario la Fe
Marina	Campins Romeu	Hospital Universitario la Fe
Isabel	Sastre Bataller	Hospital Universitario la Fe
Elena	Navarro Mocholi	Hospital Universitario la Fe
Pilar	Sánchez Alonso	Hospital Puerta de Hierro
Sabela	Novo Ponte	Hospital Puerta de Hierro
Elisa	Gamo González	Hospital Puerta de Hierro
Raquel	Martín García	Hospital Puerta de Hierro
Pablo	Mir	Hospital Universitario Virgen del Rocío
Laura	Muñoz Delgado	Hospital Universitario Virgen del Rocío
Astrid Daniela	Adarmes Gómez	Hospital Universitario Virgen del Rocío
Elena	Ojeda Lepe	Hospital Universitario Virgen del Rocío
Silvia	Jesús Maestre	Hospital Universitario Virgen del Rocío
Daniel	Macías García	Hospital Universitario Virgen del Rocío
Fátima	Carrillo García	Hospital Universitario Virgen del Rocío
Ana María	Castellano Guerrero ^2^	Hospital Universitario Virgen del Rocío
Manuela	San Eufrasio Martínez ^2^	Hospital Universitario Virgen del Rocío
Cristina	Pérez Calvo ^2^	Hospital Universitario Virgen del Rocío
Andrés	García Herruzo	Hospital Universitario Virgen del Rocío
Cristina	Gómez Rapela ^3^	Hospital Universitario Virgen del Rocío
Lorena	Garrote Espina ^3^	Hospital Universitario Virgen del Rocío
Natalia	González Rojas ^3^	Hospital Universitario Virgen del Rocío
Marta	Blázquez Estrada	Hospital Universitario Central de Asturias
Esther	Suárez San Martín	Hospital Universitario Central de Asturias
Ciara	García Fernández	Hospital Universitario Central de Asturias
Patricia	Prendes Fernández ^4^	Hospital Universitario Central de Asturias
Pilar	Tartiere ^4^	Hospital Universitario Central de Asturias
Pedro	García Ruíz Espiga	Hospital Fundación Jiménez Díaz
Cici	Feliz Feliz	Hospital Fundación Jiménez Díaz
Raúl	Espinosa Rosso	Hospital Universitario de Jerez
Lara	Mauri Fabrega	Hospital Universitario de Jerez
Marina	Mata Álvarez-Santullano	Hospital Universitario Infanta Sofía
Juan Carlos	Gómez Esteban	Hospital de Cruces
Tamara	Fernández Valle	Hospital de Cruces
Marta	Ruiz López	Hospital de Cruces
Beatriz	Tijero Merino	Hospital de Cruces
Lydia	López Manzanares	Hospital Universitario La Princesa
Inés	Muro	Hospital Universitario La Princesa
Elena	Casas Peña	Hospital Universitario La Princesa
Pablo	Lorenzo Barreto	Hospital Universitario La Princesa
Emma	Cañada Lahoz	Hospital Universitario La Princesa
Sara	Lozano Veiga	Hospital Universitario La Princesa
Maria Pilar	Gil Villar	Hospital Universitari Arnau de Vilanova
Agustín	Sancho Saldana	Hospital Universitari Arnau de Vilanova
Laura	Quibus Requena	Hospital Universitari Arnau de Vilanova
Víctor	Gómez Mayordomo	Hospitales Vithas
Berta	Solano Vila	Hospital Josep Trueta de Girona y Hospital Santa Caterina de Salt
Anna	Cots Foraster	Hospital Josep Trueta de Girona y Hospital Santa Caterina de Salt
Daniel	López Dominguez	Hospital Josep Trueta de Girona y Hospital Santa Caterina de Salt
Lilian	Vivas Villacampa	Hospital Josep Trueta de Girona y Hospital Santa Caterina de Salt
Jessica	González Ardura	Hospital de Cabueñes
Belén	Castaño García	Hospital de Cabueñes
Antonio	Sánchez Rodríguez	Hospital de Cabueñes
Sonia	Escalante Arroyo	Hospital Virgen de la Cinta
Marcos Alfredo	Mandrá	Hospital Virgen de la Cinta
Tania	Delgado Ballestero	Parc Taulí
Débora María	Cerdán Santacruz	Hospital General de Segovia
Amelia	Mendoza Rodríguez	Hospital General de Segovia
Ana	Castrillo Sanz	Hospital General de Segovia
Lorena	Caballero Sánchez	Hospital General de Segovia
Claudia	Gómez López de San Román	Hospital General de Segovia
Gustavo	Fernández-Pajarín	Complejo Hospitalario Universitario de Santiago de Compostela
Ángela	Monterde Ortega	Hospital Universitario Joan XXIII
Susana	Cantarero Duque	Hospital Universitario de Móstoles
Guillermo	González-Ortega	Hospital Universitario de Móstoles
Silvia	Martí Martínez	Hospital General Universitario Alicante
Lydia	Vela	Hospital Fundación de Alcorcón
Javier	Ruíz Martínez	Hospital Universitario Donostia
Elisabet	Mondragón Rezola	Hospital Universitario Donostia
Ana	Vinagre Aragón	Hospital Universitario Donostia
Lara	Pardina Vilella	Hospital Universitario Donostia
David	Campo Caballero	Hospital Universitario Donostia
Roberto	López Blanco	Hospital Universitario Severo Ochoa, Leganés, Madrid

The researchers are listed by center in chronological order according to the date they confirmed their participation in the project.

All investigators are neurologists except those ones with the following symbols: ^1^ SN (specialized nurse); ^2^ graduate in Biology (she will be responsible for remote data monitoring); ^3^ neuropsychologist; ^4^ study coordinator.
