# Supplementary material for: Sex Differences in the Treatment of People with Parkinson’s Disease with a Device-Aided Therapy: A Prospective Real-World Study
Source: Med Sci (Basel). 2026 Apr 27;14(2):217. doi: 10.3390/medsci14020217 (PMC13214779; doi:10.3390/medsci14020217)
Supplement: Supplementary file 1 [file medsci-14-00217-s001.zip › medsci-4256155-supplementary.pdf]

Table S1. Supplementary Material. MSs and NMSs used for this study.

Motor symptoms score (**MSs**) is the result of the sum of each item, from 0 (15 x 0) to 60 (15 x 4).

Daily Off time	0 (0%)	1 (1-25%)	2 (26-50%)	3 (51-75%)	4 (76-100%)
Daily dyskinesia time	0 (0%)	1 (1-25%)	2 (26-50%)	3 (51-75%)	4 (76-100%)
Dyskinesia severity	0 (none)	1 (slight)	2 (moderate)	3 (severe)	4 (very severe)
Painful dyskinesia	0 (none)	1 (slight)	2 (moderate)	3 (severe)	4 (very severe)
Nocturnal akinesia	0 (none)	1 (slight)	2 (moderate)	3 (severe)	4 (very severe)
Morning akinesia	0 (none)	1 (slight)	2 (moderate)	3 (severe)	4 (very severe)
Morning dystonia	0 (none)	1 (slight)	2 (moderate)	3 (severe)	4 (very severe)
FOG in "Off" state	0 (none)	1 (slight)	2 (moderate)	3 (severe)	4 (very severe)
FOG in "On" state	0 (none)	1 (slight)	2 (moderate)	3 (severe)	4 (very severe)
falls	0 (none)	1 (rarely)	2 (sometimes)	3 (often)	4 (daily)
Posture	0 (none)	1 (slight)	2 (moderate)	3 (severe)	4 (very severe)
Tremor in "Off" state	0 (none)	1 (slight)	2 (moderate)	3 (severe)	4 (very severe)
Tremor in "On" state	0 (none)	1 (slight)	2 (moderate)	3 (severe)	4 (very severe)
Hypomimia	0 (none)	1 (slight)	2 (moderate)	3 (severe)	4 (very severe)
Speech problems	0 (none)	1 (slight)	2 (moderate)	3 (severe)	4 (very severe)

FOG, freezing of gait.

Non-motor symptoms score (**NMSs**) is the result of the sum of each item, from 0 (20 x 0) to 80 (20 x 4).

Visual hallucinations	0 (none)	1 (slight)	2 (moderate)	3 (severe)	4 (very severe)
Psychosis	0 (none)	1 (slight)	2 (moderate)	3 (severe)	4 (very severe)
Impulse control disorder	0 (none)	1 (slight)	2 (moderate)	3 (severe)	4 (very severe)
Depression	0 (none)	1 (slight)	2 (moderate)	3 (severe)	4 (very severe)
Anxiety	0 (none)	1 (slight)	2 (moderate)	3 (severe)	4 (very severe)
Apathy	0 (none)	1 (slight)	2 (moderate)	3 (severe)	4 (very severe)
REM behavior disorder	0 (none)	1 (slight)	2 (moderate)	3 (severe)	4 (very severe)
Restless legs	0 (none)	1 (slight)	2 (moderate)	3 (severe)	4 (very severe)
Falling asleep insomnia	0 (none)	1 (slight)	2 (moderate)	3 (severe)	4 (very severe)
Maintenance insomnia	0 (none)	1 (slight)	2 (moderate)	3 (severe)	4 (very severe)
Diurnal somnolence	0 (none)	1 (slight)	2 (moderate)	3 (severe)	4 (very severe)
Nocturia	0 (none)	1 (slight)	2 (moderate)	3 (severe)	4 (very severe)
Other urinary symptoms	0 (none)	1 (slight)	2 (moderate)	3 (severe)	4 (very severe)
Symptomatic OH	0 (none)	1 (slight)	2 (moderate)	3 (severe)	4 (very severe)
Constipation	0 (none)	1 (slight)	2 (moderate)	3 (severe)	4 (very severe)
Sialorrhea	0 (none)	1 (slight)	2 (moderate)	3 (severe)	4 (very severe)
Dysphagia	0 (none)	1 (slight)	2 (moderate)	3 (severe)	4 (very severe)
Fatigue	0 (none)	1 (slight)	2 (moderate)	3 (severe)	4 (very severe)
Pain	0 (none)	1 (slight)	2 (moderate)	3 (severe)	4 (very severe)
Sweating	0 (none)	1 (slight)	2 (moderate)	3 (severe)	4 (very severe)

HO, orthostatic hypotension
